# Spontaneous Pneumothorax Due to Ruptured Bulla of the Azygos Lobe

**DOI:** 10.1016/j.atssr.2024.04.002

**Published:** 2024-04-19

**Authors:** Sarah E. Kim, Daniel Steeno, Alexander P. Lynch, Francis J. Podbielski

**Affiliations:** 1 Department of Surgery, University of Illinois at Chicago, Chicago, Illinois; 2 Division of Biological Sciences, Creighton University, Omaha, Nebraska; 3 Department of Thoracic Surgery, Jesse Brown Veterans Affair Medical Center, Chicago, Illinois

## Abstract

Azygos lobe is an uncommon anatomic variant that is widely recognized, but rarely associated with pneumothorax. We present a successful surgical management of a spontaneous pneumothorax resulting from rupture of a bulla in an incidentally discovered azygos lobe. The patient is a 73-year-old man who presented with the first-time occurrence of a spontaneous right pneumothorax. The patient did not tolerate nonoperative management and underwent a right thoracotomy with bullae resection in the azygos and right lower lobes for definitive management. Our treatment highlights several considerations during operative management of azygos lobe pathology.

The azygos lobe is an uncommon anatomic variant of the right upper lobe of the lung, with a reported incidence of 0.3%.^1^ The association between an azygos lobe and spontaneous pneumothorax is a rare phenomenon that has been described in only a few case reports. Our patient presented with a spontaneous pneumothorax that was found on imaging to have not only an azygos lobe, but a bulla within that lobe that was suspicious for the etiology of this nonresolving pneumothorax. We review the operative approach along with challenges faced by surgeons when addressing pathology in the azygos lobe.

This patient is a 73-year-old man with multiple medical issues including chronic obstructive pulmonary disease and prior COVID-19 infection resulting in pulmonary fibrosis with chronic hypoxic respiratory failure. He presented to the emergency department with acute worsening of his baseline dyspnea over the prior 2 weeks. Medical history is also notable for obstructive sleep apnea, heart failure, hypertension, coronary artery disease, and a remote left internal jugular deep vein thrombosis for which he is maintained on chronic apixaban. A chest radiograph in the emergency department showed a large right-sided pneumothorax with mediastinal shift (Figure 1), for which the thoracic surgery service placed a 28F chest tube with near complete reexpansion of his right lung. Unfortunately, over the next few days the patient continued to have a residual pneumothorax on his daily chest radiographs and an ongoing air leak from his chest drain. A noncontrast computed chest scan showed a small right apical pneumothorax, and bilateral reticular and ground glass opacities with bronchiectasis (consistent sequelae of his prior COVID-19 infection). He was also incidentally noted to have a right azygos fissure (Figure 2) with areas concerning for bullae in the apex of the lung. The patient did not tolerate a clamp trial of his chest tube and thus operative intervention was recommended.

**Figure 1:**
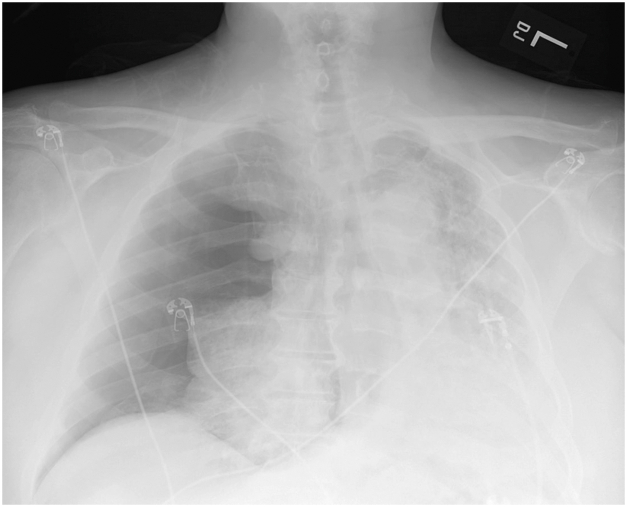
Chest radiography revealing large right pneumothorax.

**Figure 2:**
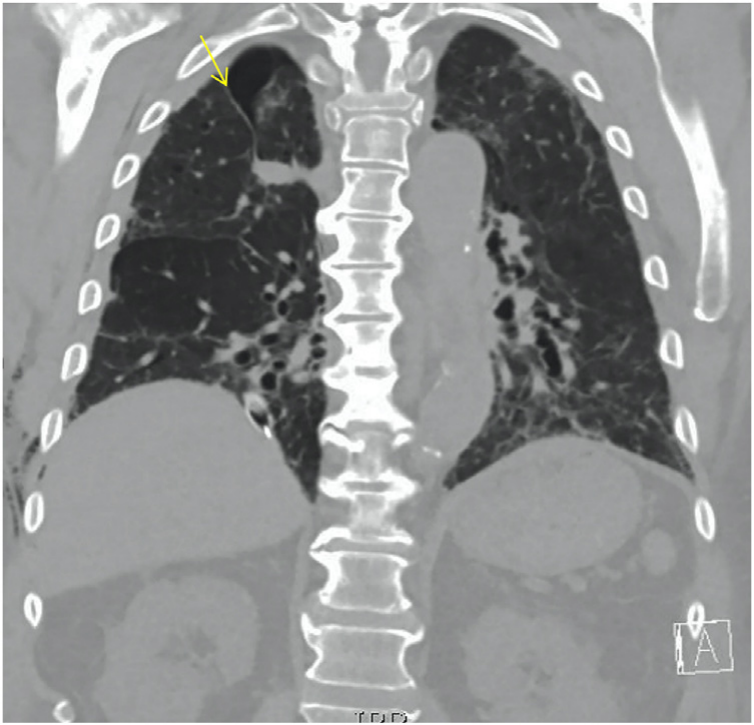
Chest computed tomography revealing several bullae and azygos fissure (arrow).

He underwent a right thoracotomy which revealed the azygos lobe. With adhesions present between the ruptured bulla and the undersurface of the chest, we were unable to deliver the azygos lobe into the operative field of view. Rather than divide the engorged and tortuous azygos vein and to maintain the existing anatomical relations we elected to create a window through the anomalous pleural reflection to expose the ruptured bulla (Figure 3). This maneuver enabled us to visualize the adhesions containing aberrant vessels and divide them with an endoscopic vascular stapler. The azygos lobe was then delivered into the operative field beneath the azygos vein. The ruptured bulla in the azygos lobe was excised in the standard fashion using an endoscopic stapler. Further exploration of the lung revealed a lower lobe bulla that was excised in a similar fashion. The patient was returned to double lung ventilation and leak test was negative. At this point, the remaining azygos lobe was gently reduced under the azygos vein to restore the preexisting anatomic configuration. The window created in the anomalous pleural reflection was closed using a running non-absorbable monofilament suture. Pleurodesis was not performed due to the patient’s preexisting extensive adhesions and parenchymal disease. Two chest tubes were placed, and the wound closed in the usual fashion. The patient was awakened and extubated in the operating room, having tolerated the procedure well.

**Figure 3:**
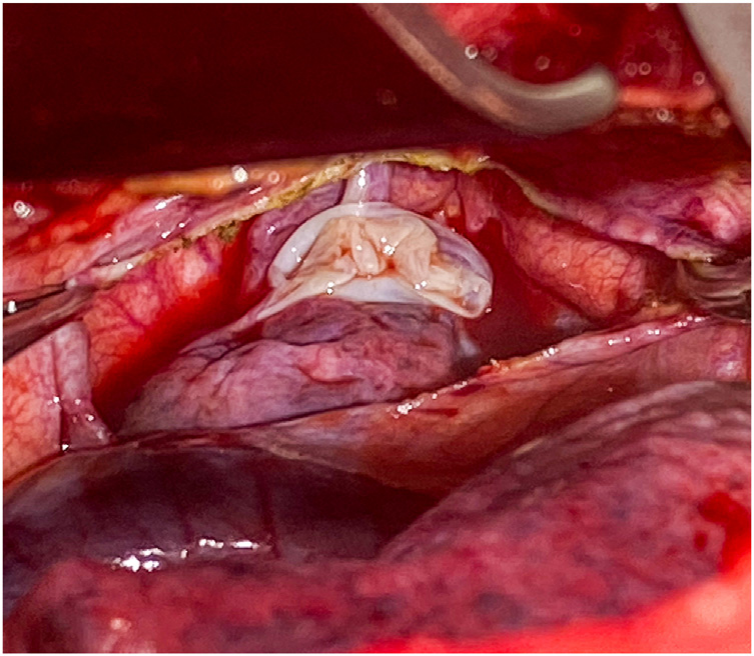
Azygos lobe with bulla with divided pleural reflection.

Over the course of the next 5 days the chest tubes were removed, and the patient discharged to home in stable condition. Postoperative follow-up visits showed excellent wound healing and complete expansion of the lung on imaging studies. Final pathology on the lung specimen showed only bullous disease with chronic inflammation and no evidence of malignancy.

## Comment

First described in 1777 by Dr Wrisberg, an azygos lobe is an anatomically separated upper lobe that is created during embryologic development when the precursor right posterior cardinal vein migrates through the right upper lobe of the lung rather than following its usual course over the apex.^2^^,^^3^ This anomalous route causes the visceral and parietal pleural layers to form the azygos fissure through the right upper lobe.

Typically, there is no clinical significance to an azygos lobe, and it is often found incidentally on imaging. It radiographically manifests itself as a fine convex line that crosses the upper lobe (as illustrated in Figure 2). The azygos lobe is not known to be associated with any lifetime increase in morbidity or mortality. From an operative standpoint, however, the surgeon is confronted with several issues when an azygos lobe is encountered, including potential variations in the venous drainage of the lung, the need for an extended lysis of adhesions to facilitate exposure, and the presence of other associated anatomical anomalies.

An extensive review of literature yields only a handful of reports of spontaneous pneumothoraces associated with an azygos lobe. Given the rarity of the incidence of coexisting spontaneous pneumothorax and azygos lobe, there has been some discussion of the potential protective effect of azygos lobe on development of pneumothorax. Proposed mechanisms include a limitation of the size of a potential pneumothorax due to adherent quality of the reflected pleura, mesoazygos decreasing mechanical strength to the apex, or the altered anatomy that intrinsically protects against bullae formation.^4^ Heretofore, there has been no proven evidence for the protective component on this anatomic variant. Our patient also had several additional underlying factors potentially predisposing him to a spontaneous pneumothorax as well as inability to heal his ruptured bulla, these include his prior smoking history, as well as pulmonary fibrosis in the setting of prior COVID-19 infection.

Finally, an azygos lobe has also been known to be associated with variations with venous drainage, including pathway variations of the azygos vein and anomalous pulmonary venous drainage. There have been cases of venous complications, such as azygos vein aneurysms/varices, which can cause rupture, thrombosis, or pulmonary embolism.^5^ Interestingly, our patient has known unprovoked left internal jugular deep vein thrombosis that was incidentally discovered several months prior, but a clear correlation between this thrombosis and the presence of an azygos lobe cannot be drawn. There are no reported or known cases of risks of upper extremity deep vein thrombosis in setting of otherwise normal azygos lobe.

In conclusion, azygos lobe is a rare anatomic variant typically of no clinical consequence and mandating no further evaluation or long-term surveillance. This case of a spontaneous pneumothorax with a ruptured bulla in the azygos lobe that required surgical intervention highlights the need for safe exposure of the lung pathology. To avoid undue blind traction on adhesions to the lung with a potential for occult bleeding, this exposure was accomplished by opening the anomalous pleural reflection rather than dividing the azygos vein. This approach also enabled us to spare the azygos vein from ligation and avoid possible venous thromboembolic complications. Our approach to this azygos bulla can be applied equally to an open, thoracoscopic, or robotic approach.
